# G**ut microbiome metagenomics in clinical practice: bridging the gap between research and precision medicine**

**DOI:** 10.1080/19490976.2025.2569739

**Published:** 2025-10-25

**Authors:** Henok Ayalew Tegegne, Tor C. Savidge

**Affiliations:** a Department of Pathology and Immunology, Baylor College of Medicine, Houston, TX, USA; b Texas Children’s Microbiome Center, Department of Pathology, Texas Children’s Hospital, Houston, TX, USA

**Keywords:** Gut microbiome, metagenomics, precision medicine, biomarkers, clinical translation, antimicrobial resistance

## Abstract

Gut microbiome metagenomics is emerging as a cornerstone of precision medicine, offering exceptional opportunities for improved diagnostics, risk stratification, and therapeutic development. Advances in high-throughput sequencing have uncovered robust microbial signatures linked to infectious, inflammatory, metabolic, and neoplastic diseases. Clinical applications now include pathogen detection, antimicrobial resistance profiling, microbiota-based therapies, and enterotype-guided patient stratification. However, translation into routine care is hindered by significant barriers including methodological variability, limited functional annotation, lack of bioinformatics standardization, and underrepresentation of global populations. This review synthesizes current translational strategies, emphasizing the need for hypothesis-driven designs, multi-omic integration, longitudinal and multi-center cohorts, and mechanistic validation. We also examine critical ethical, regulatory, and equity considerations shaping the clinical landscape. Realizing the full potential of microbiome-informed care will require globally harmonized standards, cross-sector collaboration, and inclusive frameworks that ensure scientific rigor and equitable benefit.

## Introduction

The human gut microbiome, composed of trillions of microorganisms residing in the gastrointestinal tract, plays a crucial role in maintaining health and influencing disease through metabolic, immunological, and neurological pathways.^1–3^ Increasing evidence now positions the microbiome not just as a passive symbiont but as an active regulator of host physiology, reinforcing its relevance to both foundational biology and clinical practice. Over the past decade, advancements in high-throughput sequencing technologies have revolutionized our understanding of this complex ecosystem, with metagenomics emerging as a fundamental tool to decode microbial dynamics, host–microbe interactions, and their roles in disease pathogenesis.^3^^,^^4^ This technological leap has made it possible to not only characterize microbial communities with unprecedented resolution but also to explore their clinical relevance.

Clinical interest in the gut microbiome has grown rapidly, driven by the recognition that microbial composition and function serve not only as biomarkers of disease but also as modifiable therapeutic targets.^5^^,^^6^ Metagenomic profiling now facilitates disease diagnosis, detection of antimicrobial resistance (AMR) genes, and the personalization of treatment strategies based on microbial signatures.^7^^,^^8^ For example, metagenomic analyses have identified causative pathogens in culture-negative infections and guided optimized therapies for conditions such as infective endocarditis, lower respiratory tract infections, and suspected cerebrospinal fluid infections.^9–11^ Furthermore, enterotyping, stratifying individuals by microbiome composition, adds a valuable dimension for precision diagnostics and tailored treatment selection.^12–15^

Yet, despite these advances, clinical translation of microbiome science remains limited. Key challenges, including lack of standardized protocols, inconsistent reproducibility, complex data interpretation, and regulatory barriers continue to impede integration into routine healthcare.^16–18^ Additionally, inter-individual variability, numerous uncharacterized microbial genomes, and the absence of a universally accepted definition of a “healthy” microbiome further complicate this transition. These limitations suggest that enthusiasm for clinical application must be matched with rigorous evaluation of foundational assumptions and infrastructure readiness. Recent efforts to bridge these gaps include the establishment of standardized frameworks (e.g., STORMS – STrengthening the Organization and Reporting of Microbiome Studies checklist), validated reference materials (e.g., NIST – National Institute of Standards and Technology stool reference), integrative multi-omics approaches,^17^^,^^19^ and ethical considerations.^20^ These initiatives reflect a growing consensus that progress will require more than scientific innovation, it must also involve systems-level thinking and governance. Moreover, interdisciplinary collaborations among microbiologists, clinicians, bioinformaticians, and policymakers are increasingly recognized as essential to unlocking the full clinical potential of gut microbiome metagenomics.^21^

This review critically examines the current landscape of gut microbiome metagenomics in clinical practice, highlighting promising applications, identifying persistent barriers, and proposing strategies to translate microbiome insights into precision medicine. By addressing both opportunities and challenges, we outline a roadmap to accelerate the integration of gut microbiome science into mainstream healthcare.

## Clinical application of gut microbiome metagenomics

**Figure 1.**
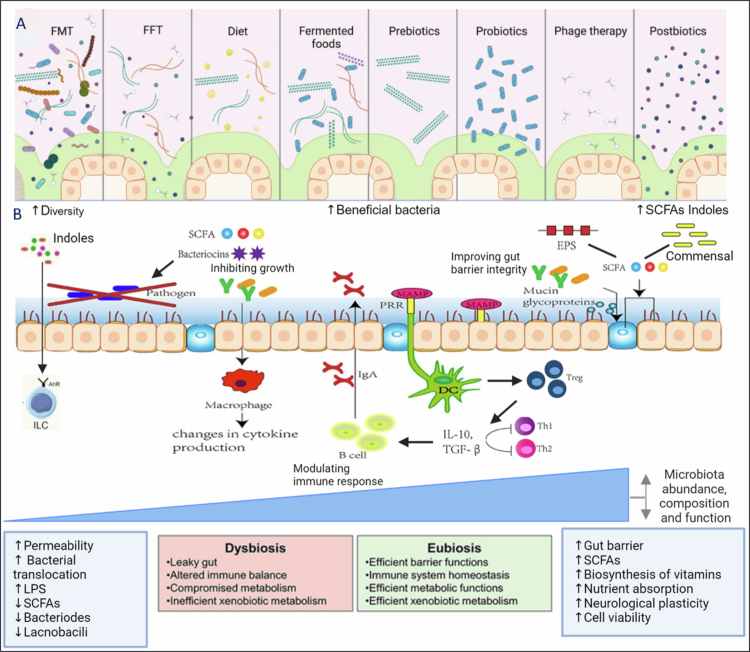
Gut modulation via FMT, FFT, diet, and microbial therapies and its impacts. (A) Depicts the modulation of the gut microbiome through interventions like Fecal Microbiota Transplantation (FMT), Fecal Filtrate Therapy (FFT), dietary adjustments (including prebiotics, probiotics, postbiotics, and fermented foods), and phage therapy. (B) Interventions are shown to alter cytokine production, leading to immune response modulation and enhanced gut barrier integrity. The figure illustrates the subsequent effects, including the increased biosynthesis of short-chain fatty acids (SCFAs) and vitamins, improved nutrient absorption, and enhanced neurological plasticity. These changes collectively contribute to maintaining cell viability and overall gastrointestinal homeostasis. Created with BioRender.com.

The rapid advancement of sequencing technologies and data analysis has propelled gut microbiome research to the forefront of precision medicine. It is no longer sufficient to view the microbiome as a passive marker; rather, comprehensive multi-omics studies underscore its active and indispensable role in disease characterization and management (Figure 1). For example, Ning et al.^22^ conducted a large-scale multi-omics integration encompassing over 1,300 metagenomes and 400 metabolomes from inflammatory bowel disease (IBD) patients and healthy controls across 13 cohorts. Their identification of consistent alterations in underreported microbial species such as *Asaccharobacter celatus*, *Gemmiger formicilis*, and *Erysipelatoclostridium ramosum*, alongside significant metabolite shifts, including amino acids, TCA-cycle intermediates, and acylcarnitines, directly links microbial community disruptions to disease status. Crucially, their construction of microbiome–metabolome correlation networks illuminated perturbed microbial pathways and functions tied to inflammation. Diagnostic models built on these multi-omics signatures achieved high accuracy (AUROC 0.92–0.98) in distinguishing IBD from controls, demonstrating the compelling clinical utility of integrated microbiome–metabolome profiling for disease diagnosis and subtype stratification.

Likewise, Qin et al.^23^ employed high-resolution serum metabolomics to profile gut microbial composition and function in type 2 diabetes (T2D). They identified 111 gut microbiota–derived metabolites significantly associated with T2D, particularly those linked to branched-chain amino acid metabolism, aromatic amino acids, and lipid pathways, which showed strong predictive power for disease progression. The diagnostic panel generated from these microbial-derived metabolites achieved an AUROC exceeding 0.80, reinforcing the potential of microbiota-informed early intervention and personalized disease management strategies.

Moreover, the power of microbiome-informed diagnostics extends beyond metabolic diseases. Zhou and Sun^24^ demonstrated this by developing a novel machine learning framework that integrates metagenomic data with clinical parameters to predict colorectal cancer (CRC) risk with superior accuracy. Their comprehensive pipeline, which unifies feature engineering, mediation analysis, statistical modeling, and network analysis, not only outperformed existing predictive methods but also highlighted key CRC-associated taxa such as elevated *Bacteroides fragilis*, shedding light on potential underlying disease mechanisms.

### Precision diagnostics and disease management

Metagenomic sequencing has decisively revolutionized infectious disease diagnostics by enabling culture-independent, sensitive, and specific pathogen detection, particularly in complex or culture-negative infections where traditional methods fail. Daquigan et al.^25^ exemplified this by integrating shotgun metagenomic sequencing with high-resolution 16S rRNA gene analysis to accurately detect *Clostridioides difficile* directly from stool samples. Their innovative approach achieved a true positive diagnostic rate exceeding 99% with minimal false positives against closely related species, underscoring its clinical reliability and potential to endorse therapeutic decision-making, although the true applicability of metagenomics to identify low abundance disease-causing pathogens with sufficient assay sensitivity and specificity requires rigorous prospective testing in the clinical microbiology laboratory.

Wilson et al.^26^ further showcased the transformative potential of metagenomics by applying unbiased metagenomic next-generation sequencing (mNGS) to cerebrospinal fluid (CSF) from patients with suspected central nervous system (CNS) infections. This method detected a broad pathogen spectrum, including bacteria, viruses, fungi, and parasites without prior assumptions, increasing diagnostic yield by 6.4% in cases where conventional testing was negative. Importantly, mNGS uncovered unexpected and rare pathogens (e.g., *Leptospira santarosai*, *Balamuthia mandrillaris*, *Taenia solium*) missed by standard microbiology, directly impacting clinical management through targeted antimicrobial or antiparasitic treatments.^27^ These studies exemplify how mNGS can overcome limitations of conventional diagnostics and enhance precision in infectious disease management.

Similarly, Wallander et al.^28^ applied 16S rRNA sequencing to diagnose bone and joint infections in patients already on antimicrobial therapy, where culture-based diagnostics often fail. Their approach improved diagnostic yield by approximately 18% over culture alone, detecting polymicrobial and fastidious organisms missed by conventional methods. This enhanced pathogen detection enabled more precise antimicrobial stewardship, reducing unnecessary broad-spectrum antibiotic use and guiding targeted therapies, demonstrating the direct clinical benefits of molecular diagnostics in complex infectious disease scenarios. In bloodstream infections, Grumaz et al.^29^ applied shotgun metagenomics directly to blood samples from critically ill patients with sepsis, achieving high diagnostic sensitivity and identifying pathogens up to 30 hours earlier than by traditional cultures. Simultaneously detecting resistance genes, their approach enabled timely, targeted antimicrobial therapy, reducing reliance on empirical treatment and improving sepsis management.

### Targeted therapeutics and treatment optimization

Metagenomics has revolutionized precision antimicrobial therapy by enabling rapid detection of AMR genes and pathogen identification directly from clinical specimens. This approach reduces the use of unnecessary broad-spectrum antibiotics, particularly important in culture-negative or polymicrobial infections, thereby improving patient outcomes and supporting antimicrobial stewardship. For example, Charalampous et al.^30^ developed a rapid 6-hour nanopore metagenomic sequencing workflow with host DNA depletion to diagnose lower respiratory bacterial infections. The method achieved 96.6% sensitivity, and 41.7% specificity compared to culture, with 100% accuracy confirmed by qPCR. It also enabled real-time identification of AMR genes, demonstrating the dual capacity for pathogen detection and resistance profiling. This facilitates early, tailored therapy adjustments and reduces reliance on empiric broad-spectrum antibiotics, advancing both patient-specific treatment and antimicrobial stewardship. Liu et al.^31^ further advanced this approach using real-time Oxford Nanopore sequencing on positive blood cultures. Their method yielded species-level pathogen identification within one hour and draft genomes within 15 hours, outperforming conventional diagnostics. Early AMR gene detection allowed timely therapy modifications, enhancing clinical decision-making and potentially improving survival.

Beyond antimicrobial selection, metagenomics critically informs personalized microbiome therapies like fecal microbiota transplantation (FMT). Wu et al.^32^ combined metagenomics and metabolomics in pediatric patients with recurrent CDI (rCDI), autism spectrum disorder (ASD) with gastrointestinal symptoms, and irritable bowel syndrome (IBS). They demonstrated that successful FMT depends on stable donor strain engraftment and restoration of key metabolites (e.g., short-chain fatty acids, bile acid derivatives, tryptophan metabolites), which support gut and immune homeostasis. The study also revealed that donor–recipient age compatibility influences these outcomes, emphasizing the potential need for age-matched donors to maximize therapeutic efficacy in children. The converse may apply to the elderly, where a youthful microbiota can exert beneficial functions over the aged microbiota. For example, Boehme et al.,^33^ demonstrated in mice that FMT from young to older animals can partially reverse age-related neuroimmune and behavioural changes, suggesting that a youthful microbiota may exert rejuvenating effects. Given that these findings are preclinical, translational studies in humans are needed, with rigorous control of donor age, diet, mucosa-associated versus luminal communities, phenocopied traits, and functional outcomes to disentangle true causal effects from confounding associations. Together, these studies suggest donor age should be tailored to the clinical goal, compatibility and metabolic stability in pediatric disease versus potential functional enhancement in the aged or age-related conditions. Optimization of clinical outcomes may also benefit from longitudinal metagenomic monitoring post-FMT to facilitate early detection of engraftment failures or adverse microbial shifts, allowing timely clinical interventions that improve patient management by offering clearer, more causally informative, and mechanistically rich insights into engraftment trajectories.^34^

### Enterotyping and predictive medicine

Enterotyping enriches personalized medicine by stratifying individuals based on dominant gut microbiome compositional patterns that influence metabolism, drug response, and disease risk. Initially proposed by Arumugam et al.,^12^ who identified three robust enterotypes (dominated by *Bacteroides*, *Prevotella*, and *Ruminococcus*) independent of age, nationality, or BMI, this concept has gained translational relevance. Wu et al.^13^ expanded enterotyping by identifying an obesity-associated enterotype dominated by *Megamonas* in a Chinese cohort of over 1,000 individuals. Functional genomics revealed *Megamonas rupellensis* degrades myo-inositol, a natural inhibitor of lipid absorption, thereby promoting lipid uptake and weight gain. Validation through in vitro intestinal organoid assays and murine models confirmed this mechanistic link, highlighting enterotype stratification’s potential to assess obesity risk and guide precision interventions.

In oncology, Zhu et al.^14^ demonstrated that distinct gut microbial communities and their metabolites modulate host response to immune checkpoint inhibitors across cancer types. Multi-omics profiling of fecal samples from 165 patients, validated in over 700 additional patients, identified five enterotypes associated with therapy response. Their predictive model integrating microbial and metabolite features robustly distinguished responders from non-responders. Notably, the metabolite phenylacetylglutamine, enriched in non-responders, impaired anti–PD1 efficacy in vivo, underscoring the functional importance of enterotype-specific microbial signatures as biomarkers guiding precision immunotherapy. Similarly, Zhu et al.^15^ reported that baseline gut microbiome enterotypes in melanoma patients predicted immune-related adverse events from immune checkpoint inhibitors. Enterotypes with *Faecalibacterium* enrichment were linked to enhanced progression-free survival but with higher adverse event risk, whereas *Bacteroides*-dominated enterotypes conferred resistance to adverse events but was associated with poorer outcomes. This dual predictive capacity highlights enterotyping as a critical tool for balancing immune-checkpoint inhibitor efficacy and safety.

### Expanding the clinical landscape

**Figure 2.**
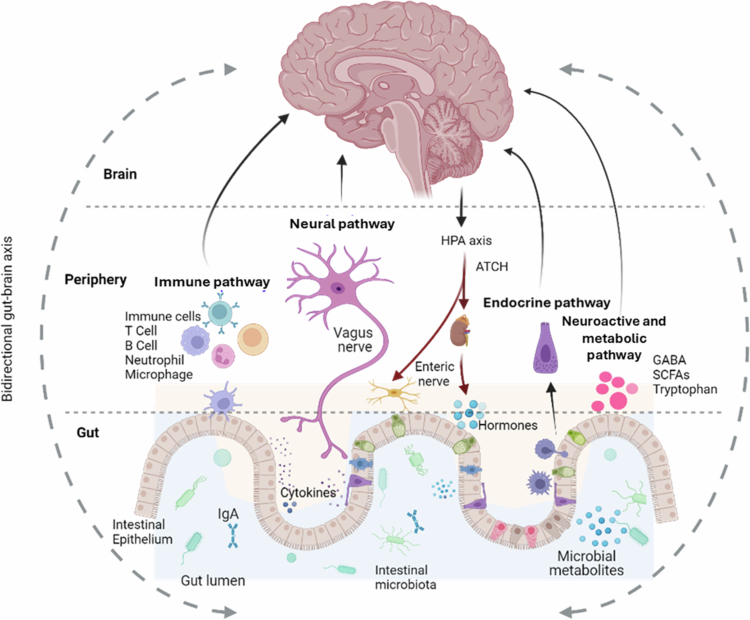
Bidirectional pathways of communication between the gut-microbiota and the brain, including immune-modulatory responses, neuronal innervation, enteroendocrine, and microbial metabolite signaling. Communication involves: (1) Immune-modulatory responses where the gut's immune activity impacts brain function and vice versa. (2) Neuronal innervation through the vagus nerve and the ENS, allowing for quick signal transmission. (3) Enteroendocrine signaling involves hormones released by gut cells affecting brain activity. (4) Microbial metabolite signaling where substances produced by gut bacteria influence brain function directly.

Gut microbiome metagenomics is rapidly expanding its clinical relevance beyond gastrointestinal and infectious diseases, now emerging as a key player in systemic conditions such as cardiovascular, neurological, and autoimmune disorders (Figure 2, Table 1). In a large-scale study published in *Cell*, Li et al.^35^ identified the gut bacterium *Oscillibacter* as a potential modulator of cholesterol metabolism. Through metagenomic and metabolomic profiling of 1,429 participants from the Framingham Heart Study, they found *Oscillibacter* to be inversely correlated with plasma and fecal cholesterol, associating with favorable lipid profiles. Multi-omic integration and in vitro validation confirmed its cholesterol-metabolizing capacity via conserved enzymatic pathways, producing less atherogenic metabolites. These findings position specific microbial taxa as both predictive biomarkers and therapeutic targets for cardiovascular intervention.

**Table 1.** Gut microbiome interconnected axes and associated diseases in human health.

Axis	Diseases
Gut-Brain Axis^5^^,^^36^^,^^37^	Anxiety Disorders
	Attention-deficit/hyperactivity disorder (ADHD)
	Bipolar disorder
	Depressive disorder
	Obsessive-compulsive disorder (OCD)
	Schizophrenia
	Alzheimer Disease (AD)
	Anorexia nervosa (AN)
	Amyotrophic Lateral Sclerosis (ALS)
	Autism spectrum disorder (ASD)
	Frontotemporal Dementia (FTD)
	Huntington's Disease (HD)
	Multiple Sclerosis (MS)
	Parkinson Disease (PD)
Gut-Heart Axis^36–38^	Atherosclerosis
	Hypertension
	Heart Failure
	Coronary Artery Disease (CAD)
	Stroke
	Peripheral Artery Disease (PAD)
Gut-Lung Axis^39^	Acute Respiratory Infections
	Mycobacterium tuberculosis
	Allergic Rhinitis
	Asthma
	Chronic Obstructive Pulmonary Disease (COPD)
	Cystic Fibrosis (CF)
	Lung Cancer
Gut-Pancreas Axis^40^	Type I diabetes
	Type II diabetes
	Obesity
Gut-Gastrointestinal Axis^32^^,^^41^^,^^42^	Colorectal Cancer
	Clostridioides difficile infection (CDI)
	Helicobacter pylori infection
	Gastroesophageal Reflux Disease (GERD)
	Gastric cancer
	Inflammatory Bowel Disease (IBD)
	Irritable Bowel Syndrome (IBS)
Gut-Liver Axis^40^^,^^42^	Alcoholic liver disease (ALD)
	Celiac Disease
	Cirrhosis
	Hepatocellular carcinoma
	Viral hepatitis
	Liver cancer
	Non-Alcoholic Fatty Liver Disease (NAFLD)
Gut-Kidney Axis^42^	Chronic kidney diseases (CKDs)
Gut-Muscle Axis	Sarcopenia
Gut–Skin Axis^42^^,^^43^	Acne Vulgaris
	Atopic Dermatitis (Eczema)
	Seborrheic Dermatitis
	Contact Dermatitis
	Hives (Urticaria)
	Psoriasis
	Rosacea
	Skin cancer
	Human papillomavirus (HPV) infection
Gut-Bone Axis^44^	Osteoporosis
Gut-Joint Axis^45^	Ankylosing Spondylitis (AS)
	Juvenile Idiopathic Arthritis (JIA)
	Osteoarthritis (OA)
	Psoriatic Arthritis
	Rheumatoid Arthritis (RA)
Gut- Lymphocytes Axis^46^	Human immunodeficiency virus (HIV)/ADIS
	Human T-cell leukemia virus (HTLV) infection

Similarly, Guan et al.^47^ demonstrated that statin therapy modulates the gut microbiota in patients with acute coronary syndrome. Using 16S rRNA sequencing and serum metabolomics, they observed that statin-treated patients exhibited enrichment of beneficial taxa (*Bifidobacterium longum*, *Anaerostipes hadrus*) and depletion of pro-inflammatory species such as *Parabacteroides merdae*. Functional predictions indicated that these compositional shifts supported lipid metabolism and anti-inflammatory activity, with rosuvastatin promoting more favorable microbial and metabolic profiles than atorvastatin. Rosuvastatin (brand name Crestor) and atorvastatin (brand names Lipitor and Atorvaliq) are lipid-lowering medications belonging to the class of HMG-CoA reductase inhibitors, commonly referred to as statins. Both agents act by inhibiting a key hepatic enzyme involved in endogenous cholesterol synthesis, thereby reducing levels of low-density lipoprotein cholesterol and triglycerides while increasing high-density lipoprotein cholesterol.^48^ These medications are widely prescribed to manage dyslipidemia, support cardiovascular risk reduction, and are often used in combination with lifestyle modifications such as diet and exercise. These results suggest that drug–microbiome interactions may also influence treatment efficacy and should be considered in cardiovascular disease management, although caution should be exercised when interpreting inferred functionality from 16S amplicon data as reported in this study.

In neurological disorders, gut microbiota alterations are increasingly recognized as contributors to disease progression. Metcalfe-Roach et al.^49^ used shotgun metagenomics to analyze stool samples from 176 Parkinson’s disease patients and 100 controls, clinical progression was followed up to 5 years. The study revealed reduced microbial diversity and connectivity, loss of carbohydrate metabolism pathways, and enrichment of proteolytic and ribosomal functions in Parkinsons’s disease patients. Notably, depletion of *Faecalibacterium prausnitzii* accounted for much of the observed functional disruption. Microbial signatures were predictive of accelerated motor symptom progression, reinforcing the gut microbiome's value as both a disease biomarker and therapeutic target in neurodegeneration.

In autism spectrum disorder, Aziz-Zadeh et al.^50^ integrated fecal metabolomics, task-based fMRI, and behavioral assessments in 43 autistic and 41 neurotypical children. They identified reductions in neuroprotective tryptophan metabolites, specifically kynurenate and indolelactate in autistic individuals. These deficits were correlated with altered neural activation in the mid-insula and mid-cingulate cortex during socio-emotional processing, which mediated the relationship between microbial metabolites and symptom severity. This study example underscores a mechanistic link between gut microbial metabolism and brain function, reinforcing the gut–brain axis in neurodevelopmental disorders.

Autoimmune diseases also demonstrate potential microbiome-mediated pathogenesis. Jiang et al.^51^ employed a two-sample Mendelian randomization design using genome-wide association study data on microbial quantitative trait loci and immune thrombocytopenic purpura risk. The analysis revealed that *Escherichia/Shigella* taxa increased immune thrombocytopenic purpura risk, while *Coprococcus* exhibited a protective effect. These findings suggest that microbial dysbiosis may causally contribute to autoimmune platelet destruction, identifying novel targets for immunomodulatory therapy. Despite these promising advances, clinical implementation remains challenged by variability in protocols, lack of methodological standardization, and complex data interpretation. Nonetheless, the breadth of associations across disease domains firmly positions gut microbiome metagenomics as a cornerstone of precision health strategies.

## Translating microbiome science into clinical practice

### Clinical relevance and study design

Successful clinical translation of microbiome science requires rigorously designed, hypothesis-driven studies with disease-specific endpoints that directly inform patient care. Longitudinal cohorts are particularly valuable because they capture temporal dynamics, control for inter-individual variability, and help distinguish cause from consequence, track disease progression or therapeutic response, and identify stable, clinically relevant biomarkers that persist across temporal fluctuations.^52^ For example, Thänert et al.^53^ conducted a comprehensive longitudinal study of 236 preterm infants, revealing that antibiotic and non-antibiotic medications significantly influenced gut microbiome composition, and that a persistent low-diversity gut microbiome was linked to later development of necrotizing enterocolitis. Similarly, Vandeputte et al.^54^ showed that in healthy adults, day-to-day intra-individual variation in gut microbial abundances often exceeded inter-individual differences, meaning cross-sectional sampling could easily miss these fluctuations or misrepresent microbial stability. These studies underscore the critical value of longitudinal designs for capturing dynamic microbial changes that single time-point studies cannot reliably detect.

To extract meaningful insights from such complex, time-resolved microbiome data, researchers can leverage statistical and computational tools designed for repeated-measures and compositional data. Approaches such as generalized linear mixed-effects models, Bayesian hierarchical models, and zero-inflated or negative binomial mixed-effects models allow robust analysis despite missing data and non-independence.^55^^,^^56^ In parallel, tools like MaAsLin2 enable feature-wise association analyses between microbial taxa and clinical or phenotypic variables while accounting for longitudinal structure, confounders, and compositional constraints.^57^ Together, these methods make it possible to uncover predictive, time-sensitive microbial markers with potential clinical relevance.^55–57^ However, while longitudinal studies provide dynamic insights into microbiome changes over time, cross-sectional studies complement this by identifying population-level microbial signatures associated with disease states. Together, these approaches enable robust biomarker discovery and inform translational pipelines for clinical application.

Notably, although longitudinal sampling may be a preferred approach to diagnose some clinical disorders, a timely diagnosis is likely to depend on cross-sectional data as repeated sampling may not be cost effective or indeed effective in some patients. When performed at the population-scale, cross-sectional studies of the gut microbiome have proven invaluable for identifying disease biomarkers and elucidating pathophysiological mechanisms. For example, Wu et al.^41^ exemplified early efforts to translate microbiome signatures into actionable diagnostics using cross-sectional data. Using 16S rRNA sequencing and machine learning, they identified microbial profiles predictive of susceptibility to CDI. By comparing stool samples from CDI patients, non-CDI diarrhea patients, and healthy controls, they found distinct taxa signatures in CDI cases, offering validated, clinically actionable biomarkers for infection risk. Franzosa et al.^58^ expanded this clinical relevance using a longitudinal, multi-omics study design to investigate IBD. Through shotgun metagenomics and untargeted LC-MS metabolomics across discovery and validation cohorts, they identified disease-specific microbial and metabolite shifts, including sphingolipid and bile acid alterations that revealed over 100 robust microbe–metabolite associations. This integrative analysis provided mechanistic insights into IBD pathophysiology and supported the development of dynamic, personalized disease monitoring strategies.

Vujkovic-Cvijin et al.^59^ reinforced the value of multi-center cohort designs. They performed metagenomic profiling across diverse populations with longitudinal monitoring of metabolic disease outcomes. The study highlighted how host confounders influence microbial signatures, emphasizing the necessity of large, heterogeneous datasets for robust and generalizable biomarker discovery. To systematically evaluate translational progress, Lee et al.^60^ proposed a structured framework for classifying genomics research across translational phases:T0 (discovery) to T2 + (public health impact). Their MEDLINE-based analysis revealed that only a small fraction of human genes progress beyond early discovery, with translational efforts concentrated in oncology and high-penetrance diseases. Applying this structured approach to microbiome science can help identify high-potential studies, guide funding and regulatory priorities, and accelerate the transition from bench to bedside. Overall, well-structured study designs and translational frameworks are indispensable for realizing the clinical potential of microbiome research.

These studies underscore the critical value of cross-sectional designs in capturing microbial features associated with diseases, which can serve as potential biomarkers for early detection and therapeutic targeting. While cross-sectional studies provide important associations, integrating these findings with longitudinal data can enhance our understanding of causality, disease progression, and mechanistic validation. Large-scale, multi-center trials are also essential to ensure reproducibility, clinical relevance, and regulatory acceptance. For example, Piccinno et al.^61^ demonstrated a model workflow in colorectal cancer research, beginning with large-scale data integration to identify microbial biomarkers, followed by hypothesis-driven validation and the development of non-invasive diagnostic tools to predict clinically meaningful outcomes.

### Validation strategies and stakeholder collaboration

Given the inherent variability in microbiome composition across individuals and populations, robust validation strategies are essential to ensure the reliability and clinical utility of microbiome-based diagnostics and therapeutics. Lee et al.^62^ illustrated this need in a large-scale, cross-cohort study of melanoma patients undergoing immune checkpoint inhibitor therapy. Despite identifying taxa such as *Akkermansia muciniphila* and *Roseburia spp.* enriched in responders, machine learning models trained on one cohort performed poorly when applied to others, demonstrating the importance of cross-validation and methodological harmonization.

Animal studies also offer critical mechanistic insight and validation. Gopalakrishnan et al.^63^ linked gut microbiome composition to anti–PD−1 therapy response in melanoma and FMT into germ-free mice confirming a causal role. Singer et al.^64^ showed neonatal gut microbiome manipulation prevents late-onset sepsis in mice, while human studies support predictive microbial biomarkers. Langelier et al.^65^ combined metagenomics and host transcriptomics to develop respiratory infection diagnostics. However, translational FMT studies in mice have several inherent limitations. Inbred specific pathogen-free and germ-free models do not fully recapitulate human physiology or co-evolved microbiomes, limiting translational relevance, as human-derived bacteria often fail to accurately phenocopy gut microbial communities typically observed in the general population. Dietary differences further contribute to this disparity, and ongoing nutritional studies using westernized diets aim to enhance the physiological relevance of such humanized microbiota models.^66^ While metagenomic approaches can improve resolution beyond taxonomy by capturing strain-level and functional variation, such methods cannot fully overcome cross-species differences, highlighting the need for cautious interpretation and validation in complementary human studies.^67^

Equally important is early and continuous collaboration with clinicians and other stakeholders to ensure research designs address real-world clinical needs. Robinson et al.^68^ demonstrated the effectiveness of this approach by involving clinicians throughout a study on *C. difficile* diagnostics. Their interdisciplinary team, including clinicians, metabolomics experts, and computational scientists used GC-MS and predictive modeling on fecal samples from 186 hospitalized patients to identify metabolic signatures that distinguished true CDI from colonization. Elevated Stickland fermentation products, metabolites produced when certain gut bacteria oxidize and reduce paired amino acids to generate energy, and depleted conjugated secondary bile acids emerged as key markers, enabling more accurate and timely diagnosis. Such cross-disciplinary, stakeholder-integrated models exemplify how rigorous validation, real-world relevance, and collaboration are essential to accelerate the translation of microbiome science into precision diagnostics and therapies.

## Technological foundations of microbiome research

### Amplicon-based 16S rRNA gene sequencing

Contemporary gut microbiome research is fundamentally shaped by advances in sequencing technologies that overcome the constraints of culture-based methods, which inadequately capture microbial diversity and non-bacterial constituents.^69^ Among these, 16S rRNA gene amplicon sequencing remains a widely utilized approach due to its cost-effectiveness and ability to survey bacterial taxa across large populations. This methodology has been instrumental in biomarker discovery and the development of personalized diagnostics.

Zhang et al.^70^ conducted a comprehensive evaluation of 16S rRNA gene amplicon sequencing across 35,889 microbial species and 157,390 samples spanning diverse environments. Employing the in silico Qscore framework, the study systematically assessed various sequencing parameters, including primer choice, platforms, pipelines, and reference databases, affirming the method’s reliability for genus-level profiling and, under optimized conditions, species-level resolution. These findings reinforce the ongoing utility of 16S sequencing in large-scale studies and its translational relevance in biomarker discovery and personalized medicine. Nevertheless, 16S rRNA sequencing presents inherent limitations. Its restricted taxonomic resolution and susceptibility to PCR amplification biases can lead to underrepresentation of key microbial groups, particularly novel or poorly characterized lineages. Eloe-Fadrosh et al.^71^ highlighted this limitation by comparing over 6,000 shotgun metagenomic assemblies to 16S datasets. They found that widely used primers failed to amplify approximately 10% of small-subunit rRNA sequences, disproportionately excluding members of candidate phyla and certain archaea, thus emphasizing the taxonomic blind spots of amplicon-based strategies.

A further limitation lies in the method’s inability to resolve closely related strains. Johnson et al.^72^ compared conventional short-read amplicon sequencing with full-length (~1,500 bp) 16S sequencing using PacBio circular consensus sequencing. Their findings demonstrated that variable region-targeted short reads (e.g., V4) were largely confined to genus-level classification, while full-length circular consensus sequencing reads, augmented by error correction and intragenomic copy phasing enabled species- and even strain-level discrimination through detection of fine-scale nucleotide variation. Complementing this, Olm et al.^73^ introduced *in Strain*, a metagenomic tool capable of identifying population microdiversity via SNP analysis of assembled genomes. This method permits high-resolution strain tracking, a capability that 16S amplicon sequencing lacks.

Recent innovations in long-read sequencing technologies have addressed several of these limitations. Full-length 16S rRNA gene sequencing via PacBio SMRT technology enables sequencing of the complete ~1,500 bp gene, in contrast to short-read protocols targeting only limited variable regions (e.g., V3–V4, ~300 bp). Buetas et al.^74^ demonstrated that full-length reads significantly enhance species-level classification accuracy (74% versus 55% with short reads), thereby allowing improved phylogenetic resolution. Lin et al.^75^ applied full-length 16S sequencing to gut microbiome profiling in metabolic-associated steatotic liver disease, showing that this approach achieved superior diagnostic accuracy (AUC = 0.87) compared to short-read methods (AUC = 0.70) when incorporated into machine learning models. These findings underscore the method’s translational potential in disease diagnostics and risk stratification.

Advances in computational analysis have further elevated the analytical utility of 16S datasets. Wu et al.^41^ developed *Taxa4Meta*, a machine learning–based meta-analysis framework designed to harmonize cross-study amplicon data by correcting for batch effects, demographic covariates, and regional primer biases. Applied across multiple cohorts, *Taxa4Meta* identified reproducible microbial signatures for CDI, IBS, and IBD, achieving high AUC values and robust cross-cohort validation. This platform exemplifies how computational harmonization can enhance existing 16S data for modern precision microbiome research.

Collectively, while 16S rRNA gene amplicon sequencing remains constrained by resolution and bias, recent innovations in long-read technologies and advanced analytical pipelines significantly extend its value. As such, this method continues to serve as a foundational tool in microbiome science, now equipped for greater taxonomic precision and cross-study comparability in both research and clinical contexts.

### Shotgun metagenomics and functional profiling

While amplicon-based sequencing has provided valuable taxonomic insights, whole-genome shotgun metagenomics (WGS) enables a more comprehensive and unbiased characterization of microbial communities. It captures not only taxonomic diversity at high resolution but also functional gene content, including metabolic pathways, virulence factors, and AMR genes.^76–78^ Bars-Cortina et al.^77^ conducted a comparative analysis of 16S rRNA and shotgun metagenomics on 156 stool samples spanning healthy individuals, patients with advanced colorectal lesions, and colorectal carcinoma cases. Shotgun metagenomics yielded higher alpha diversity, reduced data sparsity, and broader taxonomic coverage relative to 16S, while both methods identified overlapping cancer-associated signatures (e.g., *Parvimonas micra*). Importantly, shotgun data supported marginally superior machine learning classifiers, underlining the potential advantages of WGS in disease prediction. Crucially, shotgun metagenomics also facilitates direct functional profiling. Wilson et al.^79^ applied WGS to stool samples from 95 individuals (59 healthy and 36 with asthma), identifying enrichment in long-chain fatty acid biosynthesis pathways and increased richness of antibiotic resistance genes. Notably, the macrolide resistance gene *ermF* co-occurred with a *Bacteroides fragilis* toxin, illustrating the co-localization of virulence and resistance factors in disease contexts.

The clinical utility of rapid functional profiling was demonstrated by Purushothaman et al.,^76^ who optimized nanopore-based long-read WGS protocols for DNA extraction from clinical swabs. Their approach enabled real-time identification of ESKAPE pathogens and antibiotic resistance genes within hours, conferring agreement with culture-based diagnostics. Similarly, Ota et al.^80^ employed metagenomic and metatranscriptomic sequencing to profile hospital wastewater, identifying active resistance gene expression (e.g., *mexS*, *acrR*, *mexR*) and viable multidrug-resistant *Serratia nevei*, highlighting the value of functional metatranscriptomics in monitoring antibiotic resistance.

### Spatial and genome architecture mapping with Hi-C

Beyond linear sequencing, spatial genomics technologies such as Hi-Chave enabled unprecedented insights into microbial genome architecture, species interactions, and horizontal gene transfer. Hi-C, a genome-wide chromosome conformation capture method, identifies physical contacts between DNA fragments to reveal the three-dimensional organization of genomes. In microbiome research, it not only resolves genome architecture but also links plasmids and phages to their microbial hosts, detects horizontal gene transfer events, and enhances the recovery of metagenome-assembled genomes (MAGs) and host–plasmid–phage networks in complex microbiomes.^81^^,^^82^

Ivanova et al.^81^ applied Hi-C–guided metagenomic assemblies to fecal samples from ICU patients, demonstrating superior genome binning compared to conventional methods. They successfully reconstructed MAGs with integrated resistance plasmids, including *Klebsiella pneumoniae*, and identified horizontal transfer networks involving phages and plasmids, an achievement unattainable with standard shotgun approaches. Further advances in single-cell Hi-C and refined signal-to-noise correction have enabled high-confidence assignment of antimicrobial resistance genes to specific taxa within complex microbial communities. McCallum et al.^82^ utilized *meta3C* and a novel filtering pipeline—removing low-confidence interactions and repetitive elements, to assign 87 resistance genes to distinct microbial hosts, including 27 previously undescribed resistance genes. Notably, many of these resistance elements were associated with anaerobic gut commensals, reaffirming their role as key reservoirs of resistance.

These findings demonstrate how spatial metagenomics transforms our understanding of microbial ecology and antimicrobial gene resistance dynamics. By preserving physical proximity signals within DNA molecules, Hi-C enables linkage of mobile genetic elements to their hosts, thereby providing actionable insights into resistance dissemination and microbial genome evolution.

## Barriers to clinical integration

### Methodological standardization

One of the foremost challenges to integrating microbiome science into clinical practice is the absence of standardized methodologies across critical stages, ranging from sample collection and storage to DNA extraction, sequencing, and bioinformatics analysis. Variability at any of these junctures can significantly distort microbial profiles, thereby compromising cross-study comparability and undermining the clinical reliability of findings.^83^ Bharti and Grimm^16^ systematically reviewed sources of technical bias in both 16S rRNA and shotgun metagenomic workflows, highlighting disparities in preservation strategies (e.g., −80 °C freezing versus chemical stabilizers), DNA extraction techniques (mechanical versus enzymatic lysis), sequencing platforms, and analytical pipelines. To mitigate these inconsistencies, they proposed a set of best-practice guidelines, including the use of validated stabilizers, standardized bead-beating protocols for lysis, and transparent, reproducible bioinformatics workflows, each of which is essential for enhancing reproducibility and enabling clinical translation.

Efforts to establish benchmarking frameworks have been advanced through initiatives such as the Human Gut Microbiome Reference Material (RM 8048), developed by the National Institute of Standards and Technology (NIST). RM 8048 comprises rigorously characterized stool samples from both omnivorous and vegetarian donors and has undergone comprehensive multi-omic profiling, identifying over 150 microbial species and key metabolites while ensuring long-term stability and inter-batch homogeneity.^19^ Interlaboratory validation has positioned RM 8048 as a gold standard for harmonizing protocols across the microbiome research continuum.

Complementing such material standards, the STORMS checklist offers a structured framework for reporting methodologies in human microbiome research.^17^ Developed by a multidisciplinary consortium, this 18-item checklist fills gaps left by existing guidelines (e.g., STROBE, MIxS, CONSORT) by incorporating microbiome-specific considerations, including contamination controls, extraction protocols, and computational workflows. Broad adoption of STORMS facilitates transparency, reproducibility, and methodological harmonization across studies.

The Integrative Human Microbiome Project (iHMP) further exemplifies the impact of rigorous standardization. By applying harmonized protocols across multi-omic layers, metagenomics, metatranscriptomics, proteomics, and metabolomics, iHMP generated over 42 TB of longitudinal data from cohorts with IBD, preterm birth, and prediabetes.^84^ These standardized approaches enabled discoveries demonstrating that functional and strain-level microbial features provided greater predictive power for disease states than taxonomy alone. Building on this foundation, Vangay et al.^85^ convened a national workshop under the National Microbiome Data Collaborative to define standardized metadata fields, explicitly accounting for confounders such as diet, medication, and handling procedures. Most recently, Zass et al.^86^ introduced the Microbiome Research Data Toolkit, a MIxS- and PhenX-compliant resource that enables structured, harmonized metadata capture, thereby enhancing data completeness and inter-study comparability.

### Bioinformatics and functional annotation

Microbiome datasets present formidable statistical challenges due to their compositional, sparse, and high-dimensional nature. Nearing et al.^87^ benchmarked 14 differential abundance tools across 38 16S rRNA datasets, revealing substantial discrepancies in output across methods. Tools such as ALDEx2 and ANCOM-II were identified as more reliable, underscoring the need for compositionally aware and empirically validated statistical approaches. These findings highlight the value of consensus-based strategies to bolster reproducibility and analytical confidence.

Effective integration of microbiome data with clinical phenotypes and complementary omic layers, such as host transcriptomics and metabolomics, demands sophisticated computational methodologies. Yet, a persistent barrier remains: the functional annotation of microbial genes is limited, often constraining analyses to taxonomic classifications rather than mechanistic insights. The iHMP's landmark study by Huttenhower et al.^88^ addressed this challenge by combining longitudinal multi-omic data, including metagenomics, metatranscriptomics, metabolomics, and host transcriptomics in IBD cohorts. This integrative approach revealed correlations between microbial gene expression, host immune activity, and clinical outcomes, identifying dynamic, disease-specific microbial functions. Nevertheless, the authors emphasized the enduring gap in microbial gene annotation, calling for targeted efforts to close this knowledge deficit.

Beyond limited gene catalogs, current pathway annotations are often incomplete, biased toward well-studied genes, and treated as static, universal maps. This lack of context obscures tissue-, time-, and disease-specific dynamics, hindering causal inference and multi-omic integration.^89^ Context-aware annotations that incorporate directionality, temporal change, and cross-omic interactions can transform pathways into mechanistic models, revealing stable, disease-relevant perturbations that generalize across patients and support causal inference.^90^

Taxonomy-based classifications from direct sequence-based identification provide robust genus-level profiles that reduce noise and improve reproducibility, yielding more stable community classifications than inferred pathway analyses, which are often confounded by incomplete annotations and intra-genus variability.^91^ Specifically, MetaPhlAn 3 demonstrated improved accuracy in estimating microbial relative abundances at the genus level, enhancing reliability, though it masks strain-specific variation crucial for pathway prediction. As Liao et al.^92^ highlighted, different *E. coli* strains vary in virulence and metabolic capabilities, meaning genus-level annotations alone cannot capture clinically relevant differences. Strain-level, annotation-agnostic tools such as StrainScan detect biologically meaningful variation and estimate relative abundances more precisely, enabling discovery of novel functional traits beyond existing databases. Inferred pathway analyses like PICRUSt remain limited by incomplete annotations and intra-genus variability.^93^ Improved strain-specific annotation of metabolic functions, virulence factors, and drug resistance can transform descriptive analyses into predictive, clinically actionable tools,^94^ uncovering mechanistic drivers such as signaling dysregulation to enable finer patient stratification, better therapeutic prediction, and anticipation of disease evolution, making pathways more actionable than taxonomy alone.^95^

Advanced modeling strategies offer promising avenues for overcoming current analytical constraints. Ruiz-Perez et al.^96^ demonstrated the utility of microbial network inference, Bayesian approaches, and machine learning in capturing host–microbe and metabolic interactions. Building on this, Kodikara and Lê Cao^97^ introduced LUPINE, a longitudinal microbial network inference framework that incorporates time-aware graphical models to capture subject-specific effects and temporal autocorrelation. When applied to dietary intervention datasets, LUPINE elucidated transient keystone taxa and temporally dynamic microbial shifts, offering mechanistic insights into microbial community behavior over time.

Despite the promise of multi-omics integration, substantial bioinformatic and statistical hurdles persist. Challenges include aligning datasets with differing temporal and spatial resolutions, handling missing or zero-inflated values, and harmonizing feature granularity across omic platforms (e.g., genes versus metabolites). The lack of standardization in preprocessing, normalization, and batch correction further complicates integration. Moreover, high-dimensional, multi-modal models while powerful are computationally intensive and susceptible to overfitting, limiting their scalability in clinical settings.

Computational resource demands constitute an additional constraint. Large-scale, longitudinal studies employing shotgun metagenomics, untargeted metabolomics, and host transcriptomics produce heterogeneous, high-volume datasets due to repeated sampling across individuals, amplifying demands on high-performance computing, data storage, and metadata harmonization. These designs also introduce challenges such as missing data, inter-individual variability, and complex temporal correlation structures, all of which complicate reproducibility and clinical interpretation. Addressing these issues requires optimized analytical pipelines and robust integrative methods. Approaches such as multi-block partial least squares regression, canonical correlation analysis, and deep learning frameworks like MOFA +^98^ show potential but require further validation for clinical deployment.

Nonetheless, successful integration of multi-omics data remains essential for elucidating microbiome-mediated disease mechanisms and identifying robust biomarkers and therapeutic targets. Developing scalable, interpretable, and clinically validated analytical frameworks will be crucial to shifting from descriptive taxonomic profiles to functional, mechanistically grounded precision diagnostics.

Commercial gut microbiome platforms now complement research approaches through distinct methods. In 16S rRNA profiling, Biomesight adds FASTQ uploads and cross-platform comparisons, while Eurofins Genomics offers CAP/CLIA-validated clinical assays. For whole-genome metagenomics, Microba integrates a proprietary “Healthy Reference Index” and BiomeFX targets U.S. clinical practitioners. Viome uniquely applies large-scale metatranscriptomics to capture active gene expression, and CosmosID pairs rapid strain-level resolution with curated functional databases. At the frontier, GutID and PacBio achieve near-complete genome assembly and strain-level profiling, while Phase Genomics uses metagenome Hi-C to link plasmids, phages and antimicrobial-resistance genes to host strains. These platforms vary in resolution and scope, offering compositional profiles, diversity metrics, functional pathway predictions, metabolite capacity, or AI-driven dietary guidance, thereby democratizing access to microbiome insights. However, recent evaluations highlight substantial analytical variability and limitations in clinical validation. An expert consensus statement in The Lancet Gastroenterology & Hepatology proposed standardized guidelines for testing methodologies, reporting, and clinical utility while cautioning against unregulated direct-to-consumer use.^99^ Similarly, a data-driven analysis in Science using NIST reference material demonstrated wide discrepancies across commercial providers, emphasizing the need for cautious interpretation in healthcare contexts.^100^

### Ethical, regulatory, and equity challenges

As microbiome science advances toward clinical implementation, it brings forth complex ethical, regulatory, and equity considerations. Foremost among these is the issue of privacy, as individual-specific microbial signatures similar to genomic identifiers can compromise participant anonymity.^101^^,^^102^ Large-scale initiatives like the American Gut Project have underscored the difficulties surrounding data stewardship and secondary use, advocating for dynamic consent models and biobanking policies that uphold participant autonomy while enabling responsible data sharing.^20^ Without clear ethical frameworks, public trust in microbiome-informed medicine may be jeopardized, impeding clinical uptake.

Regulatory infrastructures have likewise struggled to adapt to the rapid evolution of microbiome therapeutics. The case of FMT for rCDI illustrates both the promise and pitfalls of microbial interventions. Despite robust evidence of efficacy, inconsistent practices in donor screening and preparation have raised safety concerns.^103^^,^^104^ Although the U.S. Food and Drug Administration (FDA) has begun to issue guidance, achieving global regulatory coherence will require coordinated efforts through international bodies such as the World Health Organization (WHO) and the Organisation for Economic Co-operation and Development (OECD).

Equity considerations remain equally pressing. A disproportionate focus on Western, industrialized populations limits the global relevance of microbiome research and risks exacerbating health disparities.^105^ Initiatives such as the Earth Microbiome Project and the MetaSUB Consortium aim to diversify microbial reference databases,^106^ yet unequal access to funding, infrastructure, and training continues to hinder global participation. Addressing these gaps requires targeted policy interventions, capacity-building efforts, and technology transfer to ensure that underrepresented populations also benefit from microbiome-informed healthcare innovations.

In summary, the ethical, regulatory, and equity dimensions of clinical microbiome science must be proactively addressed through inclusive study designs, transparent data governance, and globally aligned regulatory frameworks. These efforts are essential to ensure that the clinical benefits of microbiome research are both trustworthy and equitably distributed.

## Future directions and recommendations

Realizing the clinical potential of gut microbiome metagenomics hinges on the development of globally representative, functionally annotated, and longitudinally integrated datasets. Foundational efforts such as the MetaHIT Consortium^12^ laid the groundwork by cataloging the European gut microbiome, but their limited geographic and ethnic scope constrains diagnostic applicability and therapeutic relevance. Moving forward, large-scale, geographically diverse initiatives are essential to capture the full breadth of human-associated microbial variation and improve generalizability across populations.

A critical unmet need remains the functional annotation of microbial genes. Despite the wealth of taxonomic data generated through 16S rRNA and shotgun metagenomic sequencing, a substantial proportion of microbial genes remain uncharacterized, limiting mechanistic insights. Multi-omics strategies, particularly those integrating metatranscriptomics, metaproteomics, and metabolomics offer a powerful means to address this gap. Franzosa et al.^58^ demonstrated the utility of such integration by linking microbial gene expression to host physiological pathways, uncovering novel disease-associated functions. Prioritizing these approaches will be key to elucidating causal relationships and identifying actionable microbial targets for diagnostics or therapy.

Clinical translation will also require deliberate investment in clinician engagement and education. Despite growing interest in microbiome-informed medicine, knowledge gaps among healthcare professionals remain a significant bottleneck. As shown by Porcari et al.,^99^ clinician familiarity with microbiome science directly influences the uptake of microbiome-based interventions in infectious disease management. Bridging this gap demands targeted curricula, evidence-based clinical guidelines, and digital decision-support tools that distill complex microbiome data into actionable insights. From an implementation perspective, incorporating frameworks from implementation science is vital to overcoming systemic barriers to adoption. The Microbiome Quality Control (MBQC) project^107^ underscored how protocol variability undermines reproducibility, reinforcing the urgency for harmonized standards across sample processing, sequencing, and analysis. In parallel, early engagement with policymakers, regulatory bodies, and industry stakeholders is critical to ensure that scientific innovation is matched with supportive ecosystems, such as reimbursement models, regulatory clarity, and ethical governance structures. Kass et al.^108^ advocate for this multidimensional alignment as a prerequisite for realizing clinical and public health impact.

## Conclusion

Gut microbiome metagenomics is positioned to transform precision medicine by enabling personalized diagnostics, prognostics, and therapeutic interventions. Yet, this promise remains contingent upon addressing substantial translational challenges, including technical standardization, functional interpretation, ethical oversight, and clinician integration. To navigate these complexities, a concerted interdisciplinary effort is essential. Collaboration among microbiologists, computational scientists, clinicians, ethicists, and policymakers will be critical to ensure methodological rigor, equitable access, and translational success. Importantly, equity must not be an afterthought; efforts to democratize microbiome research through inclusive study designs, global capacity building, and diverse population sampling are vital to prevent the exacerbation of health disparities.

Collectively, landmark studies have validated the microbiome's utility as both a disease biomarker and a modifiable therapeutic target. The field now stands at a pivotal juncture: the shift from discovery to implementation. Advancing this transition will require not only robust science but also institutional readiness and public trust. The future of microbiome-informed healthcare lies in the integration of high-resolution microbial data with clinically relevant outcomes, translated through scalable, reproducible, and ethically guided frameworks. If achieved, this vision holds the potential to reshape medical practice by delivering more individualized, predictive, and equitable care.
